# Editorial: Innovative biocontrol strategies to manage crop and pest diseases

**DOI:** 10.3389/fmicb.2022.1052027

**Published:** 2022-10-21

**Authors:** Florence Fontaine, Ana Sofia Duarte, Jochen Fischer

**Affiliations:** ^1^Research Unit Résistance Induite et Bioprotection des Plantes RIBP EA 4707, INRAE USC 1488, University of Reims Champagne-Ardenne, Reims, France; ^2^Universidade Católica Portuguesa, Faculty of Dental Medicine, Center for Interdisciplinary Research in Health (CIIS), Viseu, Portugal; ^3^Institut für Biotechnologie und Wirkstoff-Forschung gGmbH (IBWF), Mainz, Germany

**Keywords:** crop protection, biocontrol agent (BCA), fungi, bacteria, sustainability

One of the main issues of human life will always be the efficient supply of food. During the last century a shift in agricultural practice made it possible to reduce the amount of labor to feed humanity drastically. But the mechanization of agriculture could not prevent and diminish crop losses due to plant diseases significantly (whether microbial or animal) (Vermeulen et al., 2018; Chidawanyika et al., 2019).

Nevertheless, scientists helped to find solutions for crop protection during all times. In recent years, due to climate change, a more interconnected world and mass production in combination with higher demands on crop protection solutions aiming for a lower impact on the environment new challenges arise every day. Visible to everyone the global climate is changing rapidly and many effects are increasingly manifesting across all biological and environmental systems. Additionally, humans have traded and transported species for centuries, but with an increasing speed and long-range. However, in twentieth century the spreading of species reached a new magnitude and diversity of biological invasions. Biological pests are beneficiaries of both effects. The two main methods for disease control currently available in crop production are application of fungicides (or other pesticides) and the use of plant cultivars resistant or tolerant to their pest organisms. Nevertheless, both methods have differing limitations. Public concerns regarding the health and environmental effects of pesticides, as well as the development of resistant pathogenic strains to fungicides, have reduced their potential. In many important crops, all cultivars and hybrids available to the growers worldwide are susceptible to their pest organisms and genetical modifications of the cultivars are not accepted. One possible solution is the use of biocontrol agents. For that, scientists are attempting to use the current but long-standing war between microorganisms, plants, and animals to develop biological solutions useful for broad agricultural deployment.

The editors aimed for a broad overview shedding light into different corners of modern and innovative plant protection with a focus on strategies using biological approaches.

Therefore, we searched for crop protection solutions using microorganisms and their products to obtain strategies for a modern and advanced agriculture. Some of the articles focus on fungal biocontrol agents (BCAs), such as *Trichoderma* sp. by Di Marco et al., and *Penicillium* sp. by Nguyen et al., while others focus on bacteria, such as *Pseudomonas* sp. by Yang et al. and *Bacillus* sp. as studied by He et al., Leal et al., Li et al., and Zhang N. et al.. Biological control, a phenomenon based on the antagonism between microorganisms, is considered as a sustainable eco-friendly alternative way to prevent or suppress pathogens in agriculture. For example, in this Research Topic, microbial antagonistic strategies against *Neofusicoccum parvum*, an aggressive pathogen associated to Botryosphaeria dieback (BD), are presented for the protection of Chardonnay and Tempranillo (Leal et al.). The most promising biological control trials carried out on a number of fungi that antagonize plant pests and have led to the development of so-called biofungicide products, e.g., Vintec (Chervin et al.). Another promising field of research is based on organic volatile compounds (Toral et al.). In this Research Topic on biocontrol strategies to manage crop and pest diseases, the antagonistic mechanisms of soluble non-volatile bioactive compounds emitted from *Bacillus* have been studied against plant fungal diseases and promising results have been published. The publications cover a wide range of diseases from grapevine trunk diseases to rice pathogens and nematodes on both monocotyledons and dicotyledons to *Alternaria solani* in potato plants (Zhang D. et al.) and bacterial wilt of tomato caused by *Ralstonia solanacearum* (Dong et al.). Innovative approaches also include the application of newly isolated species from marine to soil environments. The included articles allow readers an overview and enable to showcase the variety of crop pests and possible biological control strategies and methods.

The editors of this special Research Topic launched by Frontiers intend to highlight modern research regarding efficient and sustainable plant protection and shed a light on possible and rising new fields in this area.

## Author contributions

FF, AD, and JF contributed to writing this Editorial of the Research Topic and approved it for publication.

## Funding

AD was supported by National Funds through FCT—Fundação para a Ciência e a Tecnologia, I.P., under the Project UIDB/04279/2020 and CEECINST/00137/2018/CP1520/CT0013. FF is the supervisor of the academic MALDIVE Chair supported by Grand Reims and URCA.

## Conflict of interest

Author JF was employed by Institut für Biotechnologie und Wirkstoff-Forschung gGmbH (IBWF). The remaining authors declare that the research was conducted in the absence of any commercial or financial relationships that could be construed as a potential conflict of interest.

## Publisher's note

All claims expressed in this article are solely those of the authors and do not necessarily represent those of their affiliated organizations, or those of the publisher, the editors and the reviewers. Any product that may be evaluated in this article, or claim that may be made by its manufacturer, is not guaranteed or endorsed by the publisher.
